# Host species, and not environment, predicts variation in blood parasite prevalence, distribution, and diversity along a humidity gradient in northern South America

**DOI:** 10.1002/ece3.3785

**Published:** 2018-03-13

**Authors:** Paulo C. Pulgarín‐R, Juan P. Gómez, Scott Robinson, Robert E. Ricklefs, Carlos Daniel Cadena

**Affiliations:** ^1^ Laboratorio de Biología Evolutiva de Vertebrados Departamento de Ciencias Biológicas Universidad de Los Andes Bogotá Colombia; ^2^ Florida Museum of Natural History University of Florida Gainesville FL USA; ^3^ Department of Biology University of Florida Gainesville FL USA; ^4^ Spatial Epidemiology and Ecology Research Laboratory Department of Geography Emerging Pathogens Institute University of Florida Gainesville FL USA; ^5^ Department of Biology University of Missouri‐St. Louis St. Louis MO USA

**Keywords:** blood parasite, dry tropical forest, *Haemoproteus*, humid tropical forest, Magdalena River Valley, *Plasmodium*, South America

## Abstract

Environmental factors strongly influence the ecology and evolution of vector‐borne infectious diseases. However, our understanding of the influence of climatic variation on host–parasite interactions in tropical systems is rudimentary. We studied five species of birds and their haemosporidian parasites (*Plasmodium* and *Haemoproteus*) at 16 sampling sites to understand how environmental heterogeneity influences patterns of parasite prevalence, distribution, and diversity across a marked gradient in water availability in northern South America. We used molecular methods to screen for parasite infections and to identify parasite lineages. To characterize spatial heterogeneity in water availability, we used weather‐station and remotely sensed climate data. We estimated parasite prevalence while accounting for spatial autocorrelation, and used a model selection approach to determine the effect of variables related to water availability and host species on prevalence. The prevalence, distribution, and lineage diversity of haemosporidian parasites varied among localities and host species, but we found no support for the hypothesis that the prevalence and diversity of parasites increase with increasing water availability. Host species and host × climate interactions had stronger effects on infection prevalence, and parasite lineages were strongly associated with particular host species. Because climatic variables had little effect on the overall prevalence and lineage diversity of haemosporidian parasites across study sites, our results suggest that independent host–parasite dynamics may influence patterns in parasitism in environmentally heterogeneous landscapes.

## 1 INTRODUCTION

Understanding the influence of environmental heterogeneity on the diversity and prevalence of parasitic organisms may provide insight into the structure of parasite assemblages and help to explain the emergence and evolution of infectious diseases (Parratt, Numminen, & Laine, 2016; Stephens et al., 2016; Van Riper, van Riper, Goff, & Laird, 1986). Because environmental conditions can limit the distribution of pathogens, their transmission and infection rates are expected to vary across environments (Patz, Graczyk, Geller, & Vittor, 2000; Wells, O'Hara, Morand, Lessard, & Ribas, 2014), leading to a mosaic of evolutionary and ecological outcomes in host–pathogen dynamics in regions with high environmental heterogeneity (Hochachka & Dhondt, 2000; Ricklefs, Soares, Ellis, & Latta, 2016; Thompson, 1999).

Birds and their haemosporidian parasites (Apicomplexa: order Haemosporida; genera *Plasmodium, Haemoproteus,* and *Leucocytozoon*) comprise a model system that can provide insight into the interaction between environment, hosts, and pathogens. Haemosporidians are vector‐borne protozoan parasites that infect many avian taxa, as well as other vertebrates, and occur naturally in most regions and habitats (Valkiūnas, 2005). Because these parasites can reduce survival, reproductive output, longevity, and individual condition of their hosts, they may impose strong selective pressures on birds (Asghar et al., 2015; Davidar & Morton, 1993; Marzal, de Lope, Navarro, & Møller, 2004). Environmental factors are known to play a fundamental role in bird–haemosporidian interactions (Loiseau et al., 2010; Sehgal, 2015). In particular, water availability is likely a key determinant of infection patterns in haemosporidians because it is critical for the development of vector larvae (Krama et al., 2015; Loaiza & Miller, 2013; Okanga, Cumming, & Hockey, 2013; Padilla, Illera, Gonzalez‐Quevedo, Villalba, & Richardson, 2017). Accordingly, the geographic distribution, transmission, and prevalence of haemosporidians are often predicted by variation in water availability over time (Cornuault et al., 2013; Hernández‐Lara, González‐García, & Santiago‐Alarcon, 2017) and space (Coon & Martin, 2013; Gonzalez‐Quevedo, Davies, & Richardson, 2014; Svensson & Ricklefs, 2009; Wood et al., 2007). However, the influence of spatial heterogeneity in water availability on haemosporidians has rarely been assessed in the wild, particularly in tropical areas with substantial temporal and spatial variation in environmental conditions (Belo, Pinheiro, Reis, Ricklefs, & Braga, 2011; Galen & Witt, 2014; Gonzalez‐Quevedo, Pabón, & Rivera‐Gutierrez, 2016; Jones, Cheviron, & Carling, 2013; Sehgal, 2015).

Environmental heterogeneity might also promote spatial turnover in haemosporidian assemblages as well as diversification by way of climatic niche divergence (Harrigan et al., 2014; Lacorte et al., 2013) if the geographic distribution of parasite lineages were restricted to particular climatic conditions or vector–host assemblages (Cornuault et al., 2013; Fecchio, Pinheiro, et al., 2017; Moens et al., 2016). However, evidence for structuring of avian haemosporidian assemblages with respect to climate in tropical areas is scarce (Galen & Witt, 2014; Moens et al., 2016).

In addition to effects of environmental variation, host ecology and host phylogenetic relatedness can account for patterns of variation in parasite prevalence and in the distribution of parasite lineages (Fallon, Bermingham, & Ricklefs, 2003; Scordato & Kardish, 2014). Specifically, some ecological traits (e.g., nest architecture, foraging strata, and length of the incubation period) or phylogenetic affinities (e.g., oscine vs. suboscine passerines) may determine variation in the probability of infection (Arriero & Møller, 2008; Lutz et al., 2015; Poulin, 1997; Ricklefs, 1992; Ricklefs et al., 2014).

An appropriate setting in which to study parasite prevalence, distribution, and diversity of haemosporidians in relation to abiotic factors and across different host species in tropical ecosystems is the Magdalena River Valley, which runs south‐to‐north between the Eastern and Central Cordilleras of the Colombian Andes over more than 2,000 km. The valley exhibits a steep climate and habitat gradient: In areas of the upper section of the valley, mean annual precipitation is 900 mm, whereas in the middle Magdalena, mean annual precipitation reaches 4,500 mm (Álvarez‐Villa, Vélez, & Poveda, 2010). Other factors potentially influencing hosts and parasites show limited variation in the valley; the maximum difference between sites in mean annual temperature is ca. 3°C, and there is little variation (maximum difference of 500 meters) in elevation (Gómez, 2016). Several species of resident birds are codistributed across the marked precipitation gradient in the Magdalena River Valley (Hilty & Brown, 1986; Sandoval‐H, Gómez, & Cadena, 2017); these species coexist in similar habitats and are relatively common through the valley, allowing one to jointly test the effects of climate and hosts on parasitism.

We studied haemosporidian parasites infecting avian hosts in the Magdalena River Valley to assess the influence of environmental heterogeneity and host identity on patterns of parasite prevalence, distribution, and diversity. In particular, our objectives were (1) to evaluate the effect of variables describing water availability on the prevalence, distribution, and diversity of haemosporidian parasites across the humidity gradient in the Magdalena River Valley and (2) to examine whether common geographic patterns of parasitism characterize widely distributed forest birds with contrasting ecologies and evolutionary histories or whether particular patterns of infection characterize different bird species. We hypothesized that the steep climate gradient in the Magdalena River Valley results in higher parasite prevalence in areas with higher water availability, regardless of host species, implying a strong effect of climatic heterogeneity on parasitism. We also tested the alternative hypothesis that host identity across the Magdalena River Valley has a greater effect on parasitism than climate variation.

## 2 MATERIALS AND METHODS

### 2.1 Field sampling

**Figure 1:**
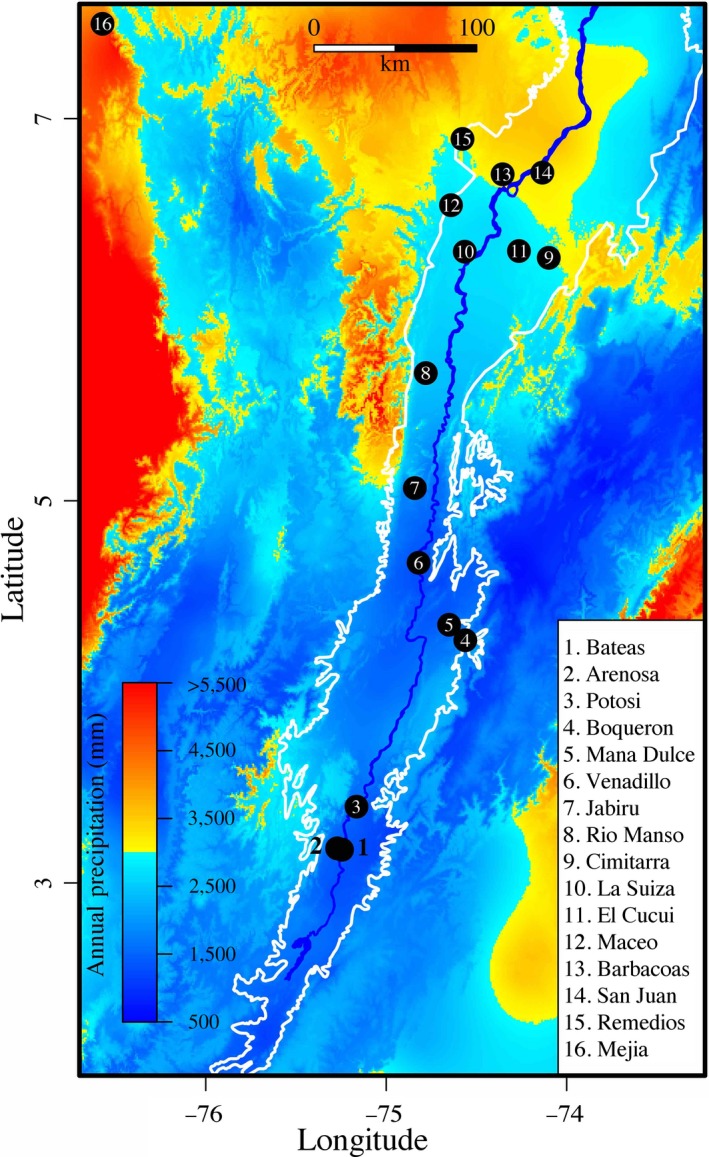
Map of central Colombia indicating the study area in the Magdalena River Valley (delineated in white; river in blue) and the Golfo de Urabá. The climatic gradient is illustrated by variation in mean annual precipitation from the WorldClim database in colors. Black circles correspond to sampled locations

We sampled birds in 15 localities distributed along the Magdalena River Valley and at one location in the Golfo de Urabá, Colombia (Figure 1). We included the latter site (La Mejía) because it allowed us to consider a study site with extremely high precipitation and because of its biotic affinity to the Magdalena River Valley (Haffer, 1967). Among our localities, variation in mean annual precipitation (range = 1,224–4,210 mm, mean = 2282.7 ± 840 mm, coefficient of variation = 36.8) and precipitation seasonality (range = 35–60, mean = 60.6 ± 23, coefficient of variation = 39.2) is substantial. In contrast, because all sites are located within the lowland tropical forest life zone (Holdridge, 1967), mean annual temperature (range = 25.4–28.1°C, mean = 27.0 ± 0.7°C, coefficient of variation = 2.6) varies minimally, and the variation does not consistently follow the northsouth direction of the Magdalena River Valley (Appendix S6).

We obtained tissue samples (pectoral muscle, liver, or heart) from specimens of five species of songbirds belonging to four families: Cocoa Woodcreeper (*Xiphorhynchus susurrans*, Furnariidae [*n *=* *46]), Ochre‐bellied Flycatcher (*Mionectes oleagineus,* Tyrannidae [*n *=* *61]), Sepia‐capped Flycatcher (*Leptopogon amaurocephalus,* Tyrannidae [*n *=* *44]), White‐bearded Manakin (*Manacus manacus*, Pipridae [*n *=* *42]), and Grey‐headed Tanager (*Eucometis penicillata*, Thraupidae [*n *=* *51]), Appendix S1). These five codistributed species are locally abundant and vary with respect to ecology and life history (Hilty & Brown, 1986). The Cocoa Woodcreeper forages on tree trunks from the understory to the forest canopy for midsized insects and occasionally small vertebrates and often follows army ant swarms (del Hoyo et al. 2003). The Ochre‐bellied Flycatcher is largely frugivorous but also eats insects and other arthropods, and usually forages in the understory but may forage up to 10 m above the ground (del Hoyo et al. 2004). The Sepia‐capped Flycatcher forages on small arthropods in shady leafy settings, usually within 8 m of the ground, and occasionally eats small fruits (del Hoyo et al. 2004). The White‐bearded Manakin is a frugivorous species that dwells in the understory, where males congregate to display at leks visited by females to select mates. Lastly, the Grey‐headed Tanager feeds mainly on fruits and insects and forages close to the ground (Isler and Isler 1999). None of the species is a long‐distance migrant and none is known to exhibit seasonal movements of tens to hundreds of kilometers across our study area (Hilty & Brown, 1986), which makes them an appropriate set of taxa in which to examine influences of the local environment on parasitism. A recent study focused on four of these species (*M. manacus* was not studied) found no discernible population genetic structure among our sampling locations in the Magdalena River Valley (Sandoval‐H et al., 2017).

Specimens were collected between June 2012 and August 2014 using mist nets located inside the forest at least 75 m from any forest edge. Due to logistical restrictions, we were unable to evenly sample the 16 localities over the same time periods; however, we obtained samples across the entire valley for both dry and wet seasons (rainfall in the region is bimodal), with an approximately balanced number of samples in different seasons (dry season: 123 individuals; wet season: 109 individuals). Although our sampling spanned 2 years, we did not obtain samples in October–December in any year. We visited half of the localities twice and half of them only once. Sampling effort across months was relatively homogeneous (mean 36.2, range 18–47 individuals per month from February to July), except for January, August, and September, during which we sampled fewer than 10 individuals. Because our sampling was not systematically designed to capture variability within sites between seasons, we accounted for the possible association between season and probability of infection using a multinomial model (Appendix S2).

For each individual, a study skin was prepared and tissue samples were preserved in 96% ethanol. Tissue samples were stored at environmental temperature for no longer than 1 month and subsequently were stored in −4°C and −80°C freezers. Specimens and tissue samples were deposited in the bird collection of the Museo de Historia Natural ANDES at Universidad de los Andes, Bogotá, Colombia (Appendix S1). All specimens were collected under permits No. 496 and No. 394 by the Ministerio de Ambiente y Desarrollo Sostenible and Agencia Nacional de Licencias Ambientales.

### 2.2 Parasite screening

We extracted whole‐genomic DNA from tissues using a dneasy blood and tissue kit according to manufacturer's instructions (Qiagen, Valencia, California). DNA quality was assessed for most individuals by amplifying a mtDNA region using avian‐specific primers (Sandoval‐H et al., 2017). We assessed infection status employing three independent PCR protocols and primers. We did not assess whether the probability of infection varied among tissues (pectoral muscle, liver, or heart), but previous work suggests this is not the case (Ramey, Fleskes, Schmutz, & Yabsley, 2013; Svensson‐Coelho et al., 2016). First, we screened all the samples for haemosporidian infections of the genera *Plasmodium* and *Haemoproteus* using primers 343F and 496R, which target a conserved 16S rRNA‐coding sequence of the parasite mtDNA spanning 154 bp (Fallon, Ricklefs, Swanson, & Bermingham, 2003). Second, we screened all positive individuals (and a subset of negatives) with a second set of primers, 3932F and DW4R, which targets a region of the cytochrome *b* gene spanning 702 bp (Olival, Stiner, & Perkins, 2007; Perkins & Schall, 2002). Third, we used the nested PCR protocols with HAEMFI‐HAEMR3‐HAEMF/HAEMR2 primers (Hellgren, Waldenström, & Bensch, 2004) to screen the same set of positive and negative individuals and to identify parasites by sequencing a 479 bp fragment of the cyt *b* gene. PCR protocols were carried out according to protocols in the aforementioned sources.

PCR products were run out on 1.5–2% agarose gels using 0.5X TBE and visualized by GelRed under ultraviolet light to check for positive infections. In all PCR, we included at least two positive controls and one negative control to confirm amplification and to check for contamination. Successful amplifications were purified and sequenced using dye‐terminator cycle‐sequencing on an ABI3500 (Life Technologies). DNA sequences were edited and aligned in geneious pro 6.1.6 with default settings. We found no evidence of mixed infections (i.e., clear double peaks in chromatograms), and all sequences were unambiguous.

### 2.3 Data analysis

#### 2.3.1 Prevalence

To examine the relationship between climate and parasite prevalence, we characterized variation in water availability across the Magdalena River Valley using four sources of climatic data including variables known to reflect water dynamics and surface hydrology at a landscape level. First, we obtained precipitation data, from 83 climatic stations across the Magdalena wet and dry forest ecoregions of Colombia, from the Instituto de Hidrología, Meteorología y Estudios Ambientales (IDEAM). Using the averages of monthly precipitation from 2011 to 2014, the period when our samples were collected, we constructed a 1 km^2^ mean monthly precipitation layer for the Magdalena Valley using a thin plate spline for interpolation (Franke, 1982). We randomly selected 20% of the data points as testing data and constructed the model with the remaining 80%. To validate the model, we calculated Pearson's correlation coefficient between the observed and predicted data and assumed that the model was accurate if the correlation coefficient exceeded 0.7. After validation, we constructed a mean annual precipitation layer by adding the values of the 12 months and calculated the mean across annual means, a precipitation seasonality layer by calculating the coefficient of variation of precipitation across months, and a layer with the precipitation in the driest quarter. Second, in addition to the short‐term data obtained from climate stations, we also characterized precipitation patterns over a longer time frame in our study sites using 50‐year means (1950–2000) from WorldClim 2.0 (Hijmans, Cameron, Parra, Jones, & Jarvis, 2005). We extracted the values of mean annual precipitation, precipitation seasonality (bio15) and precipitation of the driest quarter (bio17) from WorldClim layers. The 2011–2014 and 1950–2000 datasets were highly correlated in their measures of mean annual precipitation (*r *=* *.98) and precipitation of the driest quarter (*r *=* *.93), supporting the use of either set for our analyses. However, precipitation seasonality was less correlated between datasets (*r *=* *.42), potentially due to a strong ENSO event in 2013–2014 that caused a strong drought in the dry forest study sites located closer to the dry–wet forest boundary. After removing the three sites disproportionately affected by the ENSO event, the rest of the 2011–2014 data were also tightly correlated to the 1950–2000 data (*r *=* *.94); therefore, we used the mean annual precipitation and precipitation of the driest quarter 2011–2014 dataset for our analysis. In addition to the weather‐station data, we also characterized environmental variation using (1) a dataset describing mean annual potential evapotranspiration and aridity (Zomer, Trabucco, Bossio, & Verchot, 2008) and (2) data on mean annual cloud frequency (%) and intra‐annual cloud variability from the Global Cloud Dynamics dataset (Wilson & Jetz, 2016). All data layers were available at 1‐km^2^ resolution.

To account for potential spatial autocorrelation in our data, we performed a principal coordinates analysis using the Euclidean geographic distance among localities. We then used the first component of the principal coordinates analysis as an independent variable in model selection analyses (named “Spatial” in the models; see below). To assess whether prevalence varied among species, we considered the species identity of each individual bird as a categorical independent variable. Because of possible collinearity among climatic variables, before analyzing their influence on variation in parasite prevalence, we estimated pairwise correlations among them using Pearson's correlation coefficient. Because correlation analyses showed that several variables were highly collinear (*r *>* *.7), we retained only the following for analysis: mean annual precipitation (2011–2014 dataset), mean annual cloud frequency, intra‐annual cloud variability, and species identity (Table 1, Appendix S3). We prioritized the inclusion of mean annual precipitation over other variables when constructing models because it is likely the most direct proxy for water availability. In addition to testing for the role of raw climate variables explaining haemosporidian parasite prevalence along the Magdalena Valley, we reduced seven of the variables to three principal components (PC) serving as indices of climatic conditions. Variables were centered and their variance scaled prior to constructing the new axes. These PCs together explained 93% of the variance. The first principal component was related to mean annual precipitation (2011–2014 dataset), precipitation in the driest quarter, mean annual potential evapotranspiration, and aridity. Variables loading heavily on the second principal component were mean annual cloud frequency and precipitation seasonality, and the third principal component included variables reflecting intra‐annual variability in cloud cover (Appendix S4). We included these three components and species identity in additive models in a similar way as in analyses using raw variables.

**Table 1:** Summary of the 16 best models evaluated to explain variation in haemosporidian parasite prevalence in relation to water‐related variables across the Magdalena River Valley

Model	BIC	ΔBIC
**Species**	89.81	0.00
**Species + Clouds**	92.34	2.54
Species + Clouds.var	93.66	3.85
Species + Precipitation	93.70	3.89
Species + Clouds + Clouds.var	96.04	6.23
Species + Precipitation + Clouds	96.22	6.41
Species + Precipitation + Clouds.var	97.54	7.73
Species + Precipitation + Clouds + Clouds.var	99.94	10.14
Intercept	132.89	43.08
Precipitation	136.50	46.69
Clouds	136.64	46.83
Clouds.var	136.69	46.88
Clouds + Clouds.var	139.93	50.13
Precipitation + Clouds	140.19	50.38
Precipitation + Clouds.var	140.36	50.55
Precipitation + Clouds + Clouds.var	143.63	53.82

Models in bold face are those with strongest BIC support. Precipitation: mean annual precipitation (using the 2011–2014 dataset); Clouds: mean annual cloud frequency; Clouds.var: intra‐annual cloud variability.

Because the prevalence of haemosporidian parasites in each locality can be considered as drawn from a binomial distribution, we modeled prevalence (*p*) as a function of the environmental variables and species identity using a logit link. Because the scale of the independent variables varied across several orders of magnitude, we standardized their variation using Z‐scores. We evaluated all possible combinations of additive models and performed model selection using the Bayesian information criterion (Burnham & Anderson, 2002). We did not consider multiplicative or higher‐order models because this would require estimating too many parameters. We considered the best model to be the one with the lowest BIC, with differences of two BIC units indicating strong evidence in favor of the model with the smaller BIC (Taper & Ponciano, 2016). When ΔBIC < 2, we favored the model with the least number of parameters (i.e., we did not perform model averaging for reasons described by Bandyopadhyay, Brittan and Taper (2016), Taper and Ponciano (2016)). Confidence intervals for parameters of the best model were computed using 1,000 parametric bootstrap replicates.

To evaluate the goodness‐of‐fit of the best model, we used a likelihood ratio test (LRT) to compare fit between the selected model and a fully parameterized model. The fully parameterized model was constructed by estimating *p* for each species in each locality as the number of positive individuals of species *i* in locality *j* divided by total number of individuals sampled of species *i* in locality *j*. The LRT measures the relative likelihood of the selected model with respect to the fully parameterized model. The value of the likelihood ratio is assumed to follow a chi‐squared distribution with degrees of freedom equal to the number of parameters in the fully parameterized model minus the number of parameters of the selected model (Strong, Whipple, Child, & Dennis, 1999). If the probability of observing a value of the LRT as extreme as the one obtained is larger than 0.05, then the model selected is considered a good fit for the data (Strong et al., 1999). Second, we calculated the proportion of variance explained by a model (*R* ^2^) using the randomized quantile residuals of the relationship of the predictor variables and *p* (Dunn & Smyth, 1996). All analyses were performed in R (R Core Team 2017). Model selection was performed with package *glmulti* (Calcagno & de Mazancourt, 2010), randomized quantile residuals of GLMs were calculated using package *statmod* (Dunn & Smyth, 1996), and the Euclidean geographic distances for the PCOA were measured using package *ape* (Paradis, Claude, & Strimmer, 2004).

#### 2.3.2 Lineage diversity

We identified lineages (*Plasmodium* or *Haemoproteus*) by matching cyt *b* sequences using BLAST against sequences in GenBank and the MalAvi database (Bensch, Hellgren, & Pérez‐Tris, 2009). Sequences not matching lineages in the MalAvi database with 100% identity were regarded as new (Bensch, Pérez‐Tris, Waldenström, & Hellgren, 2004). We treated sequences as independent evolutionary lineages when they differed by ≥0.2% (1 base pair) in cyt *b* sequences (Bensch et al., 2004). Sequence divergence between lineages was calculated using Jukes–Cantor distances in geneious pro 6.1.6.

To understand the evolutionary relationships of the lineages found in the Magdalena River Valley to each other and to other lineages, we constructed phylogenetic trees using MrBayes v3.2.1 (Huelsenbeck and Ronquist, 2001) on the CIPRES Science Gateway (Miller, Pfeiffer, & Schwartz, 2010). We analyzed sequences of 322 unique lineages (*Plasmodium* [*n *=* *192] and *Haemoproteus* [*n *=* *130], including the lineages detected in our sampled areas) found in South America from the Grand Lineage Summary Table from MalAvi database (version 2.3.0). We used *Haemoproteus columbae* (GenBank accession: AF495554.1) as out‐group. Our final alignment consisted of 479 bp. Two independent MCMC analyses, each with four Metropolis‐coupled chains and default incremental heating temperature, were run for 50 million generations. We applied the default (flat Dirichlet) prior probability density in MrBayes v3.2.1. A GTR + G + I model was selected as the most appropriated model based on the BIC estimated in jModelTest 2 (Darriba, Taboada, Doallo, & Posada, 2012). Parameters and topologies were sampled every 1,000 and 50 generations, respectively. Average standard deviations of split frequencies were confirmed to be below 0.01 for each analysis indicating convergence, and 25% of the samples were discarded as burn‐in.

## 3 RESULTS

### 3.1 Prevalence and lineage diversity

**Figure 2:**
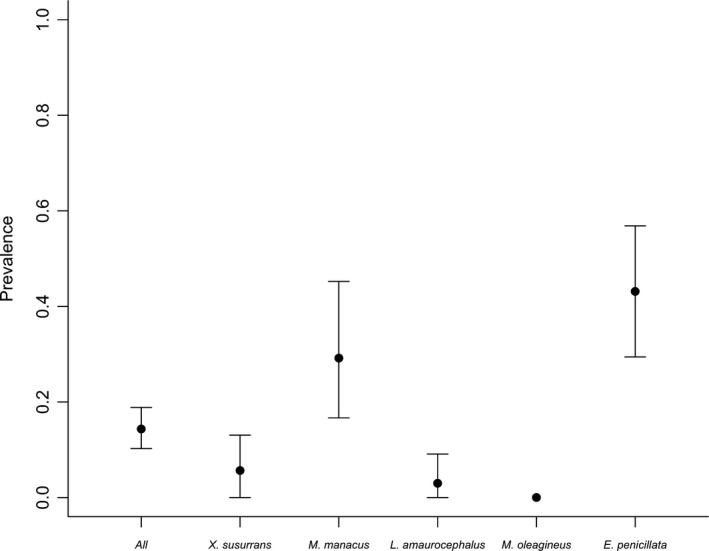
Estimated prevalence for the combined dataset and for each host species. Infection probability was higher in *Eucometis penicillata* than in any other taxon. Error bars represent 95% confidence intervals

Out of 244 individuals sampled, 35 were positive for haemosporidian infections (14.3% prevalence). However, prevalence varied substantially across host species (0–43.1%). Two species, *Eucometis penicillata* (22) and *Manacus manacus* (10), accounted for 91% of the infections, whereas *Xiphorhynchus susurrans* (2) and *Leptopogon amaurocephalus* (1) accounted for the remaining 9% (Figure 2). We found no infected individuals of *Mionectes oleagineus*. Our model testing for the effects of sampling bias in relation to climatic seasonality suggested that the probability of an individual to be infected was independent of the season in which it was sampled (Appendix S2).

**Figure 3:**
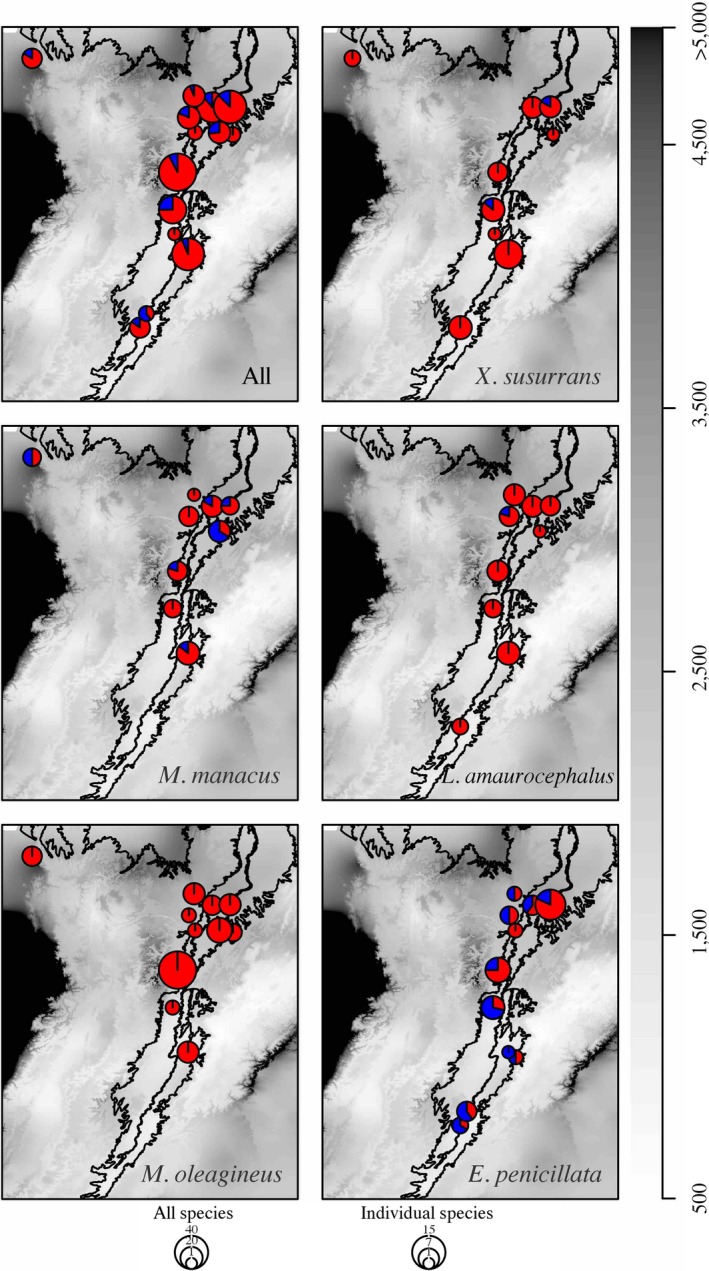
Geographic distribution of the sampled/infected birds across the study area. Pie graphs indicate the proportion of infected (in blue) and uninfected (red) individuals at each location and for each species. The gray scale indicates mean annual precipitation as in Figure 1

**Figure 4:**
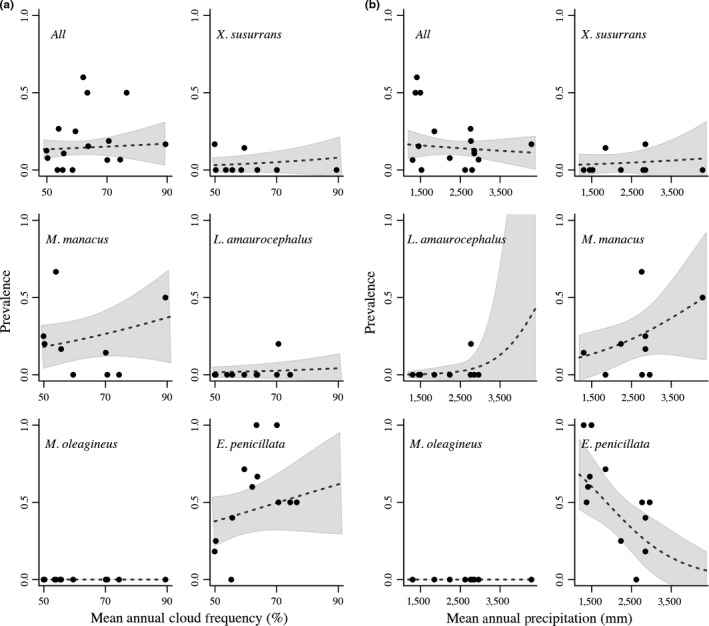
Overall prevalence was not correlated with (a) mean annual precipitation (2011–2014 dataset) or (b) mean annual cloud frequency, but varied among species. The dashed line indicates the prevalence predicted by regression models, and gray‐shaded areas are the 95% confidence interval. The dots represent the estimated prevalence data for each locality and species drawn from the predictions of binomial model

Overall prevalence varied across localities (6–30%, Figure 3), but was poorly explained by any of the climatic variables tested (e.g., mean annual precipitation, Figure 4a). The best GLM revealed that host species identity had a strong effect on variation in parasite prevalence across localities in the Magdalena River Valley; mean annual cloud frequency, cloud variability, and mean annual precipitation had weaker effects (Table 1; Figure 4b). The single best model included bird species identity and fit the data as good as the fully parameterized model; the species model accounted for more than 45% of the variance in prevalence (Table 2). The models using principal components yielded similar results to those using raw variables; the best model included only species identity as predictor of haemosporidian parasite prevalence (Appendix S5). The two species with higher prevalences showed contrasting patterns of variation in prevalence relative to mean annual precipitation: In *Eucometis penicillata*, prevalence was higher in areas with less precipitation, whereas in *Manacus manacus*, prevalence was higher in areas with more precipitation. Nonetheless, both species showed similar positive relationships between prevalence and mean annual cloud frequency (Figure 4b).

**Table 2:** Summary statistics of the best models found to explain the variation in prevalence of haemosporidian parasites across the Magdalena River Valley

Models	*Xiphorhynchus susurrans*	*Manacus manacus*	*Leptopogon amaurocephalus*	*Mionectes oleagineus*	*Eucometis penicillata*	Clouds	BICc	G^2^ (df, *p*)	*R* ^2^
Species	−2.81 (−27.55, −1.9)	−0.89 (−1.61, −0.29)	−3.48 (−27.51, −2.3)	−20.3 (NA, NA)	−0.28 (−0.83, 0.28)		89.81	35.7 (46, .86)	.47
Clouds	−2.92 (−22.06, −1.72)	−1.01 (−2.07, −0.11)	−3.59 (−22.04, −2.18)	−20.39 (NA, NA)	−0.19 (−0.78, 0.4)	0.27 (−0.18, 0.74)	92.34	34.3 (45, .88)	.6

The coefficients for each species and for mean annual cloud frequency (Clouds) are presented with their respective confidence intervals estimated using 1,000 bootstrap replicates. Because of low variability in the observations, the confidence interval for the prevalence of *Mionectes oleagineus* was not calculated. G^2^ shows the result of the likelihood ratio test between the presented model and a fully parameterized model with the degrees of freedom given by the number of parameters of the fully parameterized model minus the number of parameters of the model evaluated and the probability that G^2^ comes from a χ^2^ with degrees of freedom as shown in the table. As the number of infected individuals is assumed to be binomially distributed, the probability of success in the binomial trials (i.e., prevalences) is modeled as a logit transformation of a linear combination. The coefficients presented below are the intercept (a) and slope (β) in $\text{prevalence}=1/(1+e^{-a+\beta x})$. For example, the prevalence of *X. susurrans* in the species‐only model is $\text{prevalence}=1/(1+e^{-(-2.81)})=0.06$ and in the Clouds model is $\text{prevalence}=1/(1+e^{-(-2.92+0.27(1))})=0.07$, assuming a value of 1 for cloud cover.

We successfully sequenced 21 parasite infections: two from *Manacus manacus* and 19 from *Eucometis penicillata*; of these, 19 corresponded to *Plasmodium* and two to *Haemoproteus* (GenBank accession numbers: MG766428‐MG766448). Sequence divergence among five *Plasmodium* haplotypes ranged from 0.22 to 7.5%, whereas the two *Haemoproteus* haplotypes differed by 6.3%. Four of the haplotypes we found matched (i.e., 100% sequence identity) haemosporidian lineages reported in MalAvi or GenBank. Two of the *Plasmodium* haplotypes with a 100% match have been found in other studies, predominantly in species of oscine passerines from South and North America (e.g., *Turdus leucomelas*,* Turdus migratorius*,* Geothlypis trichas*,* Setophaga petechia, Coereba flaveola, Volatinia jacarina*, and *Cacicus cela*). The one *Haemoproteus* lineage that perfectly matched sequences in databases is also widespread and common in brush‐finches, tanagers, and cardinalids (e.g., *Tangara vassori*,* Zonotrichia capensis*,* Arremon brunneinucha*, and *Piranga olivacea*). Additionally, we discovered three novel haplotypes (Table 3). Parasite lineages were strongly associated with their hosts: Two lineages were restricted to *Manacus manacus* and five to *Eucometis penicillata*, and no lineages were shared between these two species (Table 3).

**Table 3:** Lineage distribution according to location and host in the Magdalena River Valley arranged south to north (see Figure 1)

Lineage	Locations												Species	
	1	2	3	4	5	7	8	11	12	13	14	15	*E. penicillata*	*M. manacus*
*Plasmodium* EUPE01	1											1	2	
*Plasmodium* PADOM11**	1	1			1		1			1	1		6	
*Plasmodium* EUPE02			3	1		2			1		1		8	
*Plasmodium* LEPCOR04**								1						1
*Plasmodium nucleophilum*‐DENPET03**						2							2	
*Haemoproteus coatney*i‐PIOLI03**												1	1	
*Haemoproteus* MAMA01								1						1
Totals	2	1	3	1	1	4	1	2	1	1	2	2	19	2

Haplotypes that match 100% MalAvi lineages (http://mbio-serv2.mbioekol.lu.se/Malavi/index.html) are indicated with an asterisk (**) GenBank accession numbers: MG766428‐MG766448.

Individual haemosporidian lineages varied in host breadth, prevalence, and geographic distribution, with the most host‐generalist lineages also being the most prevalent and widely distributed (PADOM11and EUPE02, Table 3). However, although these lineages were distributed across the climate gradient, most infections occurred in drier areas (Figure 3, Table 3). Three of the recovered lineages were observed only once, and two lineages only twice (Table 3). All seven lineages were distributed across the entire Magdalena River Valley (but not in La Mejía), with no particular association with sampling locations; this suggests that the climatic gradient likely has a minor influence on the spatial distribution of these parasites.

**Figure 5:**
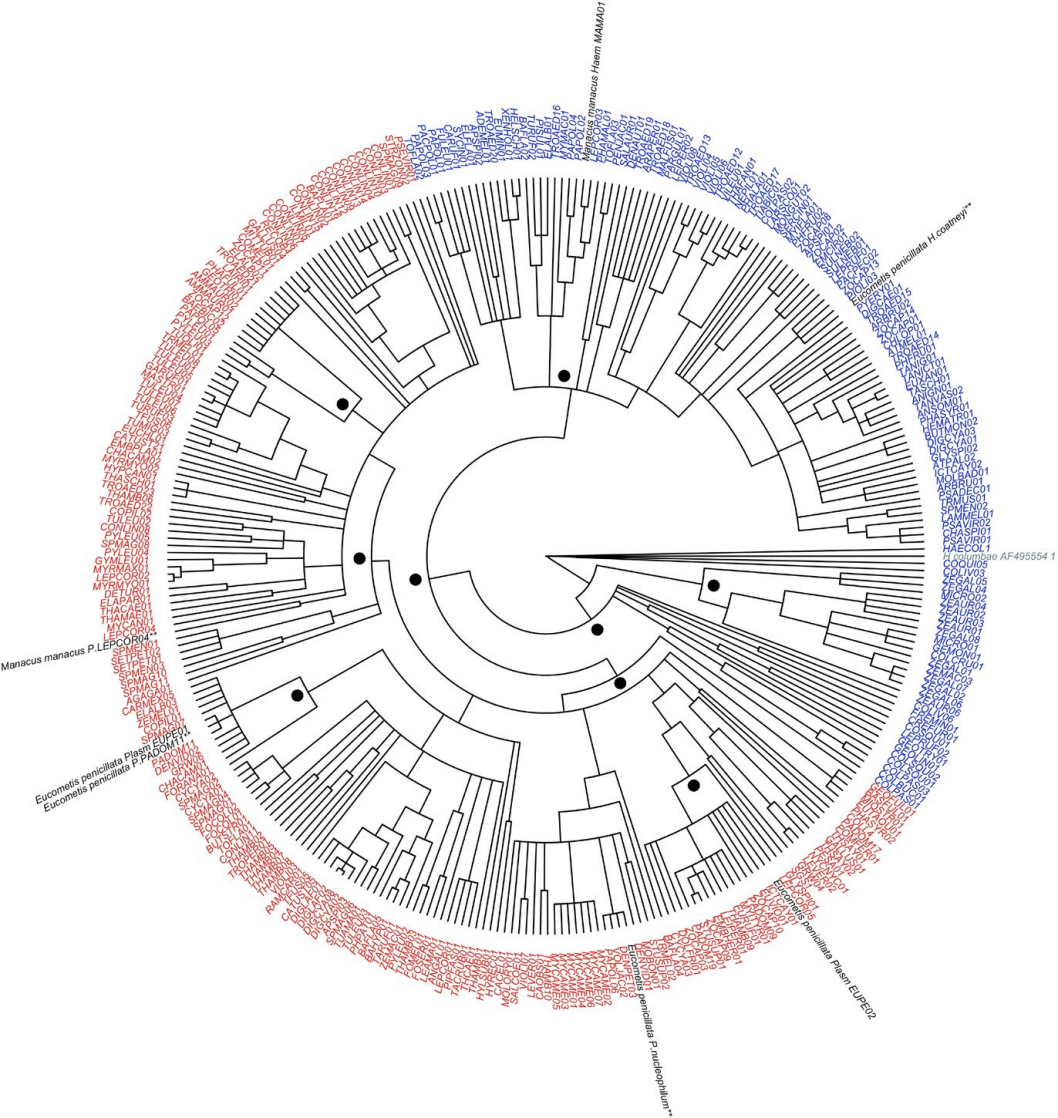
Bayesian (50% majority‐rule consensus) tree based on a 479‐bp fragment of mitochondrial cytochrome *b* gene of 322 avian haemosporidian lineages reported in South America (in both resident and migratory birds) show that lineages found in the Magdalena River Valley (shown in black, previously reported lineages in MalAvi database are denoted with asterisks) are distantly related to each other. *Plasmodium* lineages are depicted in red, *Haemoproteus* (*Parahaemoproteus*) and true *Haemoproteus* in blue including *Haemoproteus columbae* as out‐group in gray. Bayesian posterior probabilities (0.9–1.0) for main branches are shown with dots

Finally, our Bayesian phylogenetic analysis revealed that the lineages found in the Magdalena River Valley are not closely related, but belong to different clades found elsewhere, and in different species of tropical residents and migratory birds (Figure 5). Only two of the lineages we recovered (PADOM11 and EUPE01) were sisters, suggesting that the latter one might represent a local variant of the widespread and rather generalist PADOM11, which is known to occur in at least 34 species of residents and migratory birds from Canada to Uruguay.

## 4 DISCUSSION

The environmental factors influencing the emergence, spread, and evolution of haemosporidian parasites remain poorly understood, particularly in tropical areas. Although the environment is expected to strongly influence parasite prevalence, distribution, and diversity, understanding the multiple factors underlying parasitic infections in the wild remains challenging (Valkiūnas, 2005). We asked whether spatial variation in variables reflecting water availability affects haemosporidian parasite prevalence, distribution, and diversity across a steep climatic gradient in northern South America. We found no support for the hypothesis that the prevalence of haemosporidian parasites increases with water availability (as measured by several climatic variables over long‐ and short‐term temporal scales) at the landscape level in the Magdalena River Valley across a set of five species of tropical lowland birds. Likewise, we found no association of parasite diversity with climate variables, and parasite lineages were broadly distributed across sites, suggesting that water availability does not appreciably influence parasite distribution at this geographic scale. Our findings support the view that factors involved in the distribution of wildlife diseases, such as malaria, are complex and that many different factors, including ones not closely linked to environmental gradients, can influence their occurrence in host populations (Hawley & Altizer, 2011).

Consistent with our alternative hypothesis, we found that host species was a better predictor of parasite prevalence than environmental heterogeneity (i.e., variation in humidity), a result in agreement with previous findings in forest birds assemblages in insular tropical areas and in subtropical and temperate Old‐World warblers (Fallon, Bermingham, et al., 2003; Fecchio, Pinheiro, et al., 2017; Scordato & Kardish, 2014). The striking differences in infection rates among our study species may reflect variation in immunity (Bonneaud, Pérez‐Tris, Federici, Chastel, & Sorci, 2006), life history (Lutz et al., 2015; Ricklefs, 1992), or behavioral characteristics of host species (Arriero & Møller, 2008; Soares, Escudero, Penha, & Ricklefs, 2016). Most of these factors are unknown or poorly understood for our study species. However, duration of the host incubation period, which is positively associated with immune defense, has been found in other studies to be inversely related to haemosporidian parasite prevalence (Lee, Wikelski, Robinson, Robinson, & Klasing, 2008; Ricklefs, 1992). The incubation periods of the two most infected host species in the present study [*Eucometis penicillata* (14 days) and *Manacus manacus* (12 days), G. Londoño *personal communication*, (Skutch, 1954)] are shorter than those of the less infected species [*Xiphorhynchus susurrans* (18 days), *Mionectes oleagineus* (20–22 days)*, Leptopogon amaurocephalus* (19–24 days), G. Londoño com. pers, (Skutch, 1981)]. Other life history traits have been associated with parasite prevalence in birds, including nest type or height, foraging strata, survival, and flocking behavior (González et al., 2014; Lutz et al., 2015; Matthews et al., 2016), and these traits also differ between our study species (e.g., *Eucometis penicillata* and *Manacus manacus* build open‐cup nests, whereas the rest of the species nest in cavities or closed nests); other traits, including adult survival rate, are poorly known (del Hoyo, Elliott, Sargatal, & Christie, 2017).

Our results are also consistent with previous studies showing that phylogenetic background is a strong predictor of prevalence (Beadell et al., 2004; Svensson‐Coelho et al., 2013). First, the three species with lowest infection rates and longest incubation periods are all members of the same family (Tyrannidae). Likewise, the four species of suboscine passerines in our study (*Xiphorhynchus susurrans, Mionectes oleagineus, Leptopogon amaurocephalus*, and *Manacus manacus*) were less likely to be infected than the only oscine (*Eucometis penicillata*; Figure 2). This latter result is in agreement with work on temperate and tropical birds in the New World showing that oscine tanagers (Thraupidae) are more often infected than suboscine tyrant flycatchers (Tyrannidae; Fallon, Bermingham, et al., 2003; Greiner, Bennett, White, & Coombs, 1975; Ricklefs, 1992; White, Greiner, Bennett, & Herman, 1978). Addressing the hypothesis that phylogenetic affinities are predictors of parasite prevalence in our study system, however, would require greater replication of clades and species within clades.

Our results revealed nearly negligible effects of variables related to water availability on the overall prevalence and lineage diversity of haemosporidian parasites across study sites. However, the positive association between prevalence and mean annual precipitation found in *Manacus manacus* (Figure 4) provided partial support for our prediction of greater prevalence in more humid areas. In contrast, we found the opposite pattern in *Eucometis penicillata*, where most infected individuals were found in locations with lower mean annual precipitation and drier vegetation. Among the rest of the environmental variables included in the models, we detected a weak (and positive) effect of mean annual cloud frequency (%) in explaining haemosporidian prevalence, particularly for *Manacus manacus* and *Eucometis penicillata*. Although cloud dynamics are linked to water availability (e.g., affecting soil moisture, drought stress, evapotranspiration), we found that cloud cover was poorly correlated with other key environmental predictors, such as annual precipitation or precipitation seasonality (Appendix S3), signaling an independent role for cloud cover influencing host–parasite interactions. In studies of human malaria, the abundance of vectors and the probability of infection in individuals have been associated with variables such as cold‐cloud duration measured using remote sensing (Rogers, Randolph, Snow, & Hay, 2002; Sewe, Ahlm, & Rocklöv, 2016). Further work is necessary to understand how particular environmental factors modulating local water availability (e.g., wind speed or reflectance) interact with vectors and hosts to understand variation in haemosporidian prevalence and diversity in tropical birds (Norris et al., 2016; Samuel et al., 2011; Wilson & Jetz, 2016).

One limitation of this study in relation to water availability and infection patterns is the lack of information on vector ecology. Because we did not characterize the seasonal distribution, abundance, or diversity of dipteran vectors across our sampling locations, we cannot evaluate whether the precipitation gradient affected vector assemblages (Hijmans et al., 2005; Wilson & Jetz, 2016; Zomer et al., 2008), nor whether changes in vector assemblages affect parasite prevalence patterns (Glad & Crampton, 2015; Loaiza & Miller, 2013; Okanga et al., 2013; Svensson & Ricklefs, 2009). We found, however, that the proportion of individuals infected was similar between wet and dry seasons, suggesting little influence of seasonality on prevalence patterns. Likewise, although temperature has been shown to be an important predictor of prevalence across environmental gradients (Padilla et al., 2017; Zamora‐Vilchis, Williams, & Johnson, 2012), the lack of significant variation in temperature across the Magdalena River Valley (Appendix S6) suggests that, at least at the spatial scale of our study, temperature likely has similar effects on infection probability across the Magdalena River Valley. However, we acknowledge that complex interactions among temperature, water availability, and vector abundance at local scales might partly explain variability in haemosporidian prevalence in birds.

That the two most infected species exhibit contrasting patterns in the relationship between mean annual precipitation and parasite prevalence suggests independent host–parasite interactions at local scales and further indicates that patterns of infection and prevalence might be associated with local dynamics of the host or parasites (Apanius, Yorinks, Bermingham, & Ricklefs, 2000; Lachish, Knowles, Alves, Wood, & Sheldon, 2011; Sehgal, 2015). Evidence suggests that differences in prevalence between species are related to local variation in host resistance (Thompson, 2005) due to local (genetic or behavioral) adaptations to avoid or fight infections. Although populations of four of our study species (*Manacus manacus* has not been studied) are not genetically structured in the Magdalena River Valley according to one mtDNA gene (Sandoval‐H et al., 2017), variation in genes associated with parasite resistance might not mirror mtDNA variation (Sommer, 2005). One of the species in this study shows significant morphological variation along the gradient (*X. susurrans*; J. P. Gomez, unpublished data), suggesting that there might be opportunity for local adaptations despite gene flow.

Parasite specificity and distribution at local scales might also account for the observed differences in prevalence between host species (Drovetski et al., 2014; Hellgren, Pérez‐Tris, & Bensch, 2009). For instance, one *Plasmodium* lineage restricted to *Eucometis penicillata* (EUPE02) was responsible for 38% of all the infections and was present mostly in drier areas (<2,200 mm precipitation/year).

Although we still lack a comprehensive understanding of the diversity, host breadth, and geographic distribution of avian haemosporidian lineages in northern South America, our results suggest that the Magdalena River Valley likely harbors several generalist parasites because at least two *Plasmodium* lineages (PADOM 11 and *Plasmodium nucleophilum*‐DENPET03) and one *Haemoproteus* (*H. coatneyi*) infecting *Eucometis penicillata* have been found in multiple hosts and areas in the Americas, including the Antilles and North America (Durrant et al., 2006; González, Lotta, García, Moncada, & Matta, 2015; Harrigan et al., 2014; Kimura, Darbro, & Harrington, 2010; Lacorte et al., 2013; Levin et al., 2013; Marzal et al., 2011; Moens & Pérez‐Tris, 2016; Oakgrove et al., 2014; Ricklefs et al., 2016; Roos, Belo, Silveira, & Braga, 2015; Smith & Ramey, 2015). In addition, the one *Plasmodium* lineage infecting *Manacus manacus* has been previously found in another species of piprid (Blue‐crowned Manakin, *Lepidothrix coronata*) in Ecuador, Brazil, and Costa Rica (Bosholn, Fecchio, Silveira, Braga, & Anciães, 2016; Moens & Pérez‐Tris, 2016). All three haplotypes that did not match MalAvi sequences (Table 3) were closely related to lineages found in Neotropical birds of the same or closely related avian families (Beadell et al., 2006; Durrant et al., 2006; Lacorte et al., 2013). For instance, our *Haemoproteus* lineage MAMA01 (found in the manakin *Manacus manacus*) was sister to Malavi′s LEPCOR03, which has been reported in other manakins (*Lepidothrix coronata* and *Chiroxiphia pareola*), as well as in other species of suboscines (Figure 5). Unfortunately, we were not able to identify the lineages infecting *Xiphorhynchus susurrans* and *Leptopogon amaurocephalus*.

A remaining challenge after our study is to appropriately quantify the turnover of parasite assemblages across the Magdalena River Valley. We did not explicitly calculate metrics of beta diversity because of sampling issues (Beck, Holloway, & Schwanghart, 2013): The number of infected individuals varied greatly across localities and parasite composition in some localities was determined from a single infected individual or by several individuals infected with the same lineage. Therefore, more intensive sampling is required to understand the extent to which parasite assemblages might vary geographically in association with spatial variation in climate. Although we did not formally measure turnover, we believe that our data suggest parasite assemblages are likely not markedly structured by climate in our study region. This result is consistent with those of a recent study on the manakin family (Pipridae), which found no association between parasite turnover and geographic variation in climate over a broad geographic scale in the Neotropics (Fecchio, Svensson‐Coelho, et al. 2017).

Our phylogenetic analysis revealed that lineages of both *Plasmodium* and *Haemoproteus* coexisting locally belong to phylogenetically distant clades in the regional assemblage of haemosporidian lineages. This was true even for a single species; we found that *Eucometis penicillata* can host a diversity of phylogenetically distant lineages, which highlights the importance of studying host–parasite relationships in an evolutionary context (see Galen & Witt, 2014). The result of our phylogenetic analysis further suggests that haemosporidians affecting the species we studied have likely not diversified to a considerable degree within our study region, in agreement with other studies examining haemosporidian diversity in bird assemblages at local scales (Moens & Pérez‐Tris, 2016; Svensson‐Coelho et al., 2013). In addition, the spatial distributions of *Plasmodium* and *Haemoproteus* lineages do not appear to be limited by factors reflecting their evolutionary affinities at local and regional scales (Fecchio, Svensson‐Coelho, et al. 2017).

In conclusion, our study demonstrates that patterns of haemosporidian parasite prevalence, diversity, and distribution in the Magdalena River Valley mostly reflect differences among hosts, and not climate factors, such as precipitation or other variables related to water availability. Further work including a broader taxonomic spectrum at the host assemblage level and more attention to vector ecology may shed light on the ecological and evolutionary processes behind parasitic infections in wild birds across environmental gradients. In addition, work on other climatic gradients (e.g., temperature variation along elevational transects) (Ishtiaq, Bowden, & Jhala, 2017; Jones et al., 2013; Zamora‐Vilchis et al., 2012) will allow for a more comprehensive understanding of the role of environmental heterogeneity in structuring host–parasite assemblages in highly diverse tropical regions.

## DATA ACCESSIBILITY

Sequences of haemosporidian parasite lineages can be accessed with GenBank numbers: MG766428‐MG766448, and will be submitted to Malavi database (http://mbio-serv2.mbioekol.lu.se/Malavi/). Data and R code used for the analysis will be available in GitHub.

## CONFLICT OF INTEREST

None declared.

## AUTHOR CONTRIBUTIONS

PCPR, JPG, SKR, and CDC conceived the ideas and designed methodology. JPG, PCPR, SKR, CDC, and RER collected the data. PCPR and JPG analyzed the data. PCPR and CDC led the writing of the manuscript. All authors contributed critically to the drafts and gave final approval for publication.
