# Supplementary material for: Host species, and not environment, predicts variation in blood parasite prevalence, distribution, and diversity along a humidity gradient in northern South America
Source: Ecol Evol. 2018 Mar 13;8(8):3800–14. doi: 10.1002/ece3.3785 (PMC5916302; doi:10.1002/ece3.3785)

**Supporting Information**

**Appendix S1**. Information on sampling locations, species, UNIANDES-O catalog numbers (Catalog) and geographic coordinates for the 244 individuals included in this study.

| **Locality** | **Species** | **Catalog** | **Latitude** | **Longitude** |
| --- | --- | --- | --- | --- |
| Bateas | *Xiphorhynchus susurrans* | 955 | 3.17081 | -75.25124 |
| Bateas | *Xiphorhynchus susurrans* | 953 | 3.17081 | -75.25124 |
| Barbacoas | *Xiphorhynchus susurrans* | 929 | 6.71085 | -74.35172 |
| Barbacoas | *Xiphorhynchus susurrans* | 930 | 6.71085 | -74.35172 |
| Cimitarra | *Xiphorhynchus susurrans* | 945 | 6.2715 | -74.0993 |
| San Juan | *Xiphorhynchus susurrans* | 935 | 6.27975 | -74.10755 |
| La Mejía | *Xiphorhynchus susurrans* | 982 | 7.5237 | -76.58383 |
| Jabirú | *Xiphorhynchus susurrans* | 944 | 5.06656 | -74.84135 |
| Mana Dulce | *Xiphorhynchus susurrans* | 939 | 4.3512 | -74.65141 |
| Mana Dulce | *Xiphorhynchus susurrans* | 941 | 4.3512 | -74.65141 |
| Jabirú | *Xiphorhynchus susurrans* | 950 | 5.06656 | -74.84135 |
| Jabirú | *Xiphorhynchus susurrans* | 948 | 5.06656 | -74.84135 |
| Barbacoas | *Xiphorhynchus susurrans* | 926 | 6.71085 | -74.35172 |
| Río Manso | *Xiphorhynchus susurrans* | 936 | 5.66584 | -74.78178 |
| Mana Dulce | *Xiphorhynchus susurrans* | 988 | 4.3512 | -74.65141 |
| Mana Dulce | *Xiphorhynchus susurrans* | 898 | 4.3512 | -74.65141 |
| Río Manso | *Xiphorhynchus susurrans* | 1073 | 5.66584 | -74.78178 |
| Bateas | *Xiphorhynchus susurrans* | 1072 | 3.17081 | -75.25124 |
| Bateas | *Xiphorhynchus susurrans* | 1070 | 3.17081 | -75.25124 |
| Río Manso | *Xiphorhynchus susurrans* | 938 | 5.66584 | -74.78178 |
| San Juan | *Xiphorhynchus susurrans* | 934 | 6.27975 | -74.10755 |
| San Juan | *Xiphorhynchus susurrans* | 931 | 6.27975 | -74.10755 |
| Mana Dulce | *Xiphorhynchus susurrans* | 942 | 4.3512 | -74.65141 |
| Mana Dulce | *Xiphorhynchus susurrans* | 940 | 4.3512 | -74.65141 |
| Mana Dulce | *Xiphorhynchus susurrans* | 943 | 4.3512 | -74.65141 |
| Jabirú | *Xiphorhynchus susurrans* | 952 | 5.06656 | -74.84135 |
| Jabirú | *Xiphorhynchus susurrans* | 951 | 5.06656 | -74.84135 |
| Jabirú | *Xiphorhynchus susurrans* | 946 | 5.06656 | -74.84135 |
| Jabirú | *Xiphorhynchus susurrans* | 949 | 5.06656 | -74.84135 |
| Barbacoas | *Xiphorhynchus susurrans* | 927 | 6.71085 | -74.35172 |
| Barbacoas | *Xiphorhynchus susurrans* | 925 | 6.71085 | -74.35172 |
| Barbacoas | *Xiphorhynchus susurrans* | 928 | 6.71085 | -74.35172 |
| San Juan | *Xiphorhynchus susurrans* | 932 | 6.27975 | -74.10755 |
| San Juan | *Xiphorhynchus susurrans* | 933 | 6.27975 | -74.10755 |
| Río Manso | *Xiphorhynchus susurrans* | 937 | 5.66584 | -74.78178 |
| Mana Dulce | *Xiphorhynchus susurrans* | 987 | 4.3512 | -74.65141 |
| Mana Dulce | *Xiphorhynchus susurrans* | 986 | 4.3512 | -74.65141 |
| Mana Dulce | *Xiphorhynchus susurrans* | 984 | 4.3512 | -74.65141 |
| La Mejía | *Xiphorhynchus susurrans* | 985 | 7.5237 | -76.58383 |
| La Mejía | *Xiphorhynchus susurrans* | 983 | 7.5237 | -76.58383 |
| San Juan | *Xiphorhynchus susurrans* | 1075 | 6.27975 | -74.10755 |
| Río Manso | *Xiphorhynchus susurrans* | 1077 | 5.66584 | -74.78178 |
| Bateas | *Xiphorhynchus susurrans* | 1069 | 3.17081 | -75.25124 |
| Bateas | *Xiphorhynchus susurrans* | 954 | 3.17081 | -75.25124 |
| Venadillo | *Xiphorhynchus susurrans* | 947 | 4.6756 | -74.82046 |
| Bateas | *Xiphorhynchus susurrans* | 1071 | 3.17081 | -75.25124 |
| San Juan | *Mionectes oleagineus* | 971 | 6.27975 | -74.10755 |
| Barbacoas | *Mionectes oleagineus* | 831 | 6.71085 | -74.35172 |
| Barbacoas | *Mionectes oleagineus* | 830 | 6.71085 | -74.35172 |
| Barbacoas | *Mionectes oleagineus* | 823 | 6.71085 | -74.35172 |
| Maceo | *Mionectes oleagineus* | 829 | 6.55162 | -74.64276 |
| Río Manso | *Mionectes oleagineus* | 753 | 5.66584 | -74.78178 |
| Remedios | *Mionectes oleagineus* | 827 | 6.90883 | -74.57204 |
| Remedios | *Mionectes oleagineus* | 828 | 6.90883 | -74.57204 |
| Remedios | *Mionectes oleagineus* | 818 | 6.90883 | -74.57204 |
| La Mejía | *Mionectes oleagineus* | 1001 | 7.5237 | -76.58383 |
| La Suiza | *Mionectes oleagineus* | 841 | 6.3036 | -74.65141 |
| Río Manso | *Mionectes oleagineus* | 757 | 5.66584 | -74.78178 |
| Río Manso | *Mionectes oleagineus* | 754 | 5.66584 | -74.78178 |
| San Juan | *Mionectes oleagineus* | 967 | 6.27975 | -74.10755 |
| Jabirú | *Mionectes oleagineus* | 976 | 5.06656 | -74.84135 |
| Barbacoas | *Mionectes oleagineus* | 820 | 6.71085 | -74.35172 |
| Mana Dulce | *Mionectes oleagineus* | 834 | 4.3512 | -74.65141 |
| Mana Dulce | *Mionectes oleagineus* | 832 | 4.3512 | -74.65141 |
| Mana Dulce | *Mionectes oleagineus* | 836 | 4.3512 | -74.65141 |
| El Cucui | *Mionectes oleagineus* | 810 | 6.30707 | -74.26435 |
| El Cucui | *Mionectes oleagineus* | 815 | 6.30707 | -74.26435 |
| El Cucui | *Mionectes oleagineus* | 813 | 6.30707 | -74.26435 |
| Jabirú | *Mionectes oleagineus* | 826 | 5.06656 | -74.84135 |
| Maceo | *Mionectes oleagineus* | 825 | 6.55162 | -74.64276 |
| San Juan | *Mionectes oleagineus* | 816 | 6.27975 | -74.10755 |
| San Juan | *Mionectes oleagineus* | 814 | 6.27975 | -74.10755 |
| San Juan | *Mionectes oleagineus* | 812 | 6.27975 | -74.10755 |
| Remedios | *Mionectes oleagineus* | 819 | 6.90883 | -74.57204 |
| Río Manso | *Mionectes oleagineus* | 844 | 5.66584 | -74.78178 |
| Río Manso | *Mionectes oleagineus* | 839 | 5.66584 | -74.78178 |
| Río Manso | *Mionectes oleagineus* | 838 | 5.66584 | -74.78178 |
| Mana Dulce | *Mionectes oleagineus* | 1004 | 4.3512 | -74.65141 |
| Mana Dulce | *Mionectes oleagineus* | 995 | 4.3512 | -74.65141 |
| El Cucui | *Mionectes oleagineus* | 996 | 6.30707 | -74.26435 |
| El Cucui | *Mionectes oleagineus* | 1005 | 6.30707 | -74.26435 |
| El Cucui | *Mionectes oleagineus* | 994 | 6.30707 | -74.26435 |
| La Mejía | *Mionectes oleagineus* | 1003 | 7.5237 | -76.58383 |
| La Mejía | *Mionectes oleagineus* | 999 | 7.5237 | -76.58383 |
| Río Manso | *Mionectes oleagineus* | 1031 | 5.66584 | -74.78178 |
| Río Manso | *Mionectes oleagineus* | 1030 | 5.66584 | -74.78178 |
| Río Manso | *Mionectes oleagineus* | 977 | 5.66584 | -74.78178 |
| Río Manso | *Mionectes oleagineus* | 756 | 5.66584 | -74.78178 |
| Cimitarra | *Mionectes oleagineus* | 974 | 6.2715 | -74.0993 |
| Cimitarra | *Mionectes oleagineus* | 970 | 6.2715 | -74.0993 |
| Cimitarra | *Mionectes oleagineus* | 975 | 6.2715 | -74.0993 |
| La Suiza | *Mionectes oleagineus* | 842 | 6.3036 | -74.65141 |
| Barbacoas | *Mionectes oleagineus* | 821 | 6.71085 | -74.35172 |
| San Juan | *Mionectes oleagineus* | 972 | 6.27975 | -74.10755 |
| Mana Dulce | *Mionectes oleagineus* | 833 | 4.3512 | -74.65141 |
| El Cucui | *Mionectes oleagineus* | 811 | 6.30707 | -74.26435 |
| Remedios | *Mionectes oleagineus* | 817 | 6.90883 | -74.57204 |
| Remedios | *Mionectes oleagineus* | 822 | 6.90883 | -74.57204 |
| Río Manso | *Mionectes oleagineus* | 843 | 5.66584 | -74.78178 |
| Río Manso | *Mionectes oleagineus* | 840 | 5.66584 | -74.78178 |
| La Mejía | *Mionectes oleagineus* | 1000 | 7.5237 | -76.58383 |
| La Mejía | *Mionectes oleagineus* | 998 | 7.5237 | -76.58383 |
| El Cucui | *Mionectes oleagineus* | 997 | 6.30707 | -74.26435 |
| Río Manso | *Mionectes oleagineus* | 1032 | 5.66584 | -74.78178 |
| Río Manso | *Mionectes oleagineus* | 1033 | 5.66584 | -74.78178 |
| Cimitarra | *Mionectes oleagineus* | 968 | 6.2715 | -74.0993 |
| Barbacoas | *Leptopogon amaurocephalus* | 865 | 6.71085 | -74.35172 |
| Maceo | *Leptopogon amaurocephalus* | 870 | 6.55162 | -74.64276 |
| Maceo | *Leptopogon amaurocephalus* | 850 | 6.55162 | -74.64276 |
| Maceo | *Leptopogon amaurocephalus* | 860 | 6.55162 | -74.64276 |
| Arenosa | *Leptopogon amaurocephalus* | 852 | 3.18115 | -75.27187 |
| Mana Dulce | *Leptopogon amaurocephalus* | 864 | 4.3512 | -74.65141 |
| Mana Dulce | *Leptopogon amaurocephalus* | 851 | 4.3512 | -74.65141 |
| Jabirú | *Leptopogon amaurocephalus* | 869 | 5.06656 | -74.84135 |
| Barbacoas | *Leptopogon amaurocephalus* | 866 | 6.71085 | -74.35172 |
| Barbacoas | *Leptopogon amaurocephalus* | 847 | 6.71085 | -74.35172 |
| Barbacoas | *Leptopogon amaurocephalus* | 849 | 6.71085 | -74.35172 |
| Barbacoas | *Leptopogon amaurocephalus* | 846 | 6.71085 | -74.35172 |
| Barbacoas | *Leptopogon amaurocephalus* | 855 | 6.71085 | -74.35172 |
| San Juan | *Leptopogon amaurocephalus* | 871 | 6.27975 | -74.10755 |
| San Juan | *Leptopogon amaurocephalus* | 848 | 6.27975 | -74.10755 |
| San Juan | *Leptopogon amaurocephalus* | 863 | 6.27975 | -74.10755 |
| San Juan | *Leptopogon amaurocephalus* | 857 | 6.27975 | -74.10755 |
| Remedios | *Leptopogon amaurocephalus* | 867 | 6.90883 | -74.57204 |
| Remedios | *Leptopogon amaurocephalus* | 868 | 6.90883 | -74.57204 |
| Remedios | *Leptopogon amaurocephalus* | 845 | 6.90883 | -74.57204 |
| Río Manso | *Leptopogon amaurocephalus* | 861 | 5.66584 | -74.78178 |
| Río Manso | *Leptopogon amaurocephalus* | 873 | 5.66584 | -74.78178 |
| Río Manso | *Leptopogon amaurocephalus* | 854 | 5.66584 | -74.78178 |
| Mana Dulce | *Leptopogon amaurocephalus* | 990 | 4.3512 | -74.65141 |
| Mana Dulce | *Leptopogon amaurocephalus* | 993 | 4.3512 | -74.65141 |
| Mana Dulce | *Leptopogon amaurocephalus* | 992 | 4.3512 | -74.65141 |
| San Juan | *Leptopogon amaurocephalus* | 1048 | 6.27975 | -74.10755 |
| Río Manso | *Leptopogon amaurocephalus* | 1050 | 5.66584 | -74.78178 |
| Río Manso | *Leptopogon amaurocephalus* | 1046 | 5.66584 | -74.78178 |
| Bateas | *Leptopogon amaurocephalus* | 1041 | 3.17081 | -75.25124 |
| Bateas | *Leptopogon amaurocephalus* | 1040 | 3.17081 | -75.25124 |
| Bateas | *Leptopogon amaurocephalus* | 1045 | 3.17081 | -75.25124 |
| El Cucui | *Leptopogon amaurocephalus* | 853 | 6.30707 | -74.26435 |
| Jabirú | *Leptopogon amaurocephalus* | 544 | 5.06656 | -74.84135 |
| Jabirú | *Leptopogon amaurocephalus* | 859 | 5.06656 | -74.84135 |
| Mana Dulce | *Leptopogon amaurocephalus* | 991 | 4.3512 | -74.65141 |
| Jabirú | *Leptopogon amaurocephalus* | 1044 | 5.06656 | -74.84135 |
| Mana Dulce | *Leptopogon amaurocephalus* | 862 | 4.3512 | -74.65141 |
| Remedios | *Leptopogon amaurocephalus* | 1042 | 6.90883 | -74.57204 |
| Remedios | *Leptopogon amaurocephalus* | 1043 | 6.90883 | -74.57204 |
| Río Manso | *Leptopogon amaurocephalus* | 1049 | 5.66584 | -74.78178 |
| Remedios | *Leptopogon amaurocephalus* | 1047 | 6.90883 | -74.57204 |
| Barbacoas | *Manacus manacus* | 883 | 6.71085 | -74.35172 |
| Maceo | *Manacus manacus* | 876 | 6.55162 | -74.64276 |
| Remedios | *Manacus manacus* | 894 | 6.90883 | -74.57204 |
| Mana Dulce | *Manacus manacus* | 895 | 4.3512 | -74.65141 |
| Barbacoas | *Manacus manacus* | 890 | 6.71085 | -74.35172 |
| Barbacoas | *Manacus manacus* | 880 | 6.71085 | -74.35172 |
| Mana Dulce | *Manacus manacus* | 893 | 4.3512 | -74.65141 |
| Jabirú | *Manacus manacus* | 881 | 5.06656 | -74.84135 |
| Jabirú | *Manacus manacus* | 889 | 5.06656 | -74.84135 |
| Jabirú | *Manacus manacus* | 888 | 5.06656 | -74.84135 |
| Maceo | *Manacus manacus* | 897 | 6.55162 | -74.64276 |
| Maceo | *Manacus manacus* | 879 | 6.55162 | -74.64276 |
| Maceo | *Manacus manacus* | 886 | 6.55162 | -74.64276 |
| Río Manso | *Manacus manacus* | 891 | 5.66584 | -74.78178 |
| San Juan | *Manacus manacus* | 884 | 6.27975 | -74.10755 |
| Mana Dulce | *Manacus manacus* | 1008 | 4.3512 | -74.65141 |
| Mana Dulce | *Manacus manacus* | 1020 | 4.3512 | -74.65141 |
| Mana Dulce | *Manacus manacus* | 1009 | 4.3512 | -74.65141 |
| El Cucui | *Manacus manacus* | 1010 | 6.30707 | -74.26435 |
| El Cucui | *Manacus manacus* | 1007 | 6.30707 | -74.26435 |
| San Juan | *Manacus manacus* | 1039 | 6.27975 | -74.10755 |
| San Juan | *Manacus manacus* | 1037 | 6.27975 | -74.10755 |
| Barbacoas | *Manacus manacus* | 892 | 6.71085 | -74.35172 |
| Jabirú | *Manacus manacus* | 882 | 5.06656 | -74.84135 |
| Barbacoas | *Manacus manacus* | 875 | 6.71085 | -74.35172 |
| Maceo | *Manacus manacus* | 885 | 6.55162 | -74.64276 |
| Río Manso | *Manacus manacus* | 887 | 5.66584 | -74.78178 |
| Río Manso | *Manacus manacus* | 896 | 5.66584 | -74.78178 |
| Mana Dulce | *Manacus manacus* | 1018 | 4.3512 | -74.65141 |
| El Cucui | *Manacus manacus* | 1013 | 6.30707 | -74.26435 |
| El Cucui | *Manacus manacus* | 1011 | 6.30707 | -74.26435 |
| El Cucui | *Manacus manacus* | 1012 | 6.30707 | -74.26435 |
| La Mejía | *Manacus manacus* | 1029 | 7.5237 | -76.58383 |
| San Juan | *Manacus manacus* | 1038 | 6.27975 | -74.10755 |
| Río Manso | *Manacus manacus* | 1036 | 5.66584 | -74.78178 |
| La Mejía | *Manacus manacus* | 1015 | 7.5237 | -76.58383 |
| La Mejía | *Manacus manacus* | 1017 | 7.5237 | -76.58383 |
| Barbacoas | *Manacus manacus* | 878 | 6.71085 | -74.35172 |
| El Cucui | *Manacus manacus* | 1019 | 6.30707 | -74.26435 |
| Mana Dulce | *Manacus manacus* | 1006 | 4.3512 | -74.65141 |
| La Mejía | *Manacus manacus* | 1014 | 7.5237 | -76.58383 |
| Río Manso | *Manacus manacus* | 877 | 5.66584 | -74.78178 |
| Potosí | *Eucometis penicillata* | 781 | 3.40542 | -75.15874 |
| Río Manso | *Eucometis penicillata* | 800 | 5.66584 | -74.78178 |
| Barbacoas | *Eucometis penicillata* | 777 | 6.71085 | -74.35172 |
| La Suiza | *Eucometis penicillata* | 770 | 6.3036 | -74.65141 |
| Río Manso | *Eucometis penicillata* | 801 | 5.66584 | -74.78178 |
| Maceo | *Eucometis penicillata* | 804 | 6.55162 | -74.64276 |
| Maceo | *Eucometis penicillata* | 776 | 6.55162 | -74.64276 |
| Bateas | *Eucometis penicillata* | 786 | 3.17081 | -75.25124 |
| Maceo | *Eucometis penicillata* | 765 | 6.55162 | -74.64276 |
| San Juan | *Eucometis penicillata* | 790 | 6.27975 | -74.10755 |
| Jabirú | *Eucometis penicillata* | 783 | 5.06656 | -74.84135 |
| Jabirú | *Eucometis penicillata* | 782 | 5.06656 | -74.84135 |
| Jabirú | *Eucometis penicillata* | 809 | 5.06656 | -74.84135 |
| Boqueron | *Eucometis penicillata* | 792 | 4.27002 | -74.56168 |
| Bateas | *Eucometis penicillata* | 795 | 3.17081 | -75.25124 |
| Potosí | *Eucometis penicillata* | 779 | 3.40542 | -75.15874 |
| Barbacoas | *Eucometis penicillata* | 794 | 6.71085 | -74.35172 |
| La Suiza | *Eucometis penicillata* | 796 | 6.3036 | -74.65141 |
| Río Manso | *Eucometis penicillata* | 798 | 5.66584 | -74.78178 |
| San Juan | *Eucometis penicillata* | 797 | 6.27975 | -74.10755 |
| Río Manso | *Eucometis penicillata* | 799 | 5.66584 | -74.78178 |
| Jabirú | *Eucometis penicillata* | 1051 | 5.06656 | -74.84135 |
| San Juan | *Eucometis penicillata* | 1054 | 6.27975 | -74.10755 |
| San Juan | *Eucometis penicillata* | 1055 | 6.27975 | -74.10755 |
| Río Manso | *Eucometis penicillata* | 787 | 5.66584 | -74.78178 |
| Mana Dulce | *Eucometis penicillata* | 981 | 4.3512 | -74.65141 |
| Río Manso | *Eucometis penicillata* | 780 | 5.66584 | -74.78178 |
| Río Manso | *Eucometis penicillata* | 771 | 5.66584 | -74.78178 |
| Río Manso | *Eucometis penicillata* | 807 | 5.66584 | -74.78178 |
| Remedios | *Eucometis penicillata* | 793 | 6.90883 | -74.57204 |
| Remedios | *Eucometis penicillata* | 772 | 6.90883 | -74.57204 |
| Arenosa | *Eucometis penicillata* | 774 | 3.18115 | -75.27187 |
| Potosí | *Eucometis penicillata* | 789 | 3.40542 | -75.15874 |
| Barbacoas | *Eucometis penicillata* | 775 | 6.71085 | -74.35172 |
| Barbacoas | *Eucometis penicillata* | 766 | 6.71085 | -74.35172 |
| Jabirú | *Eucometis penicillata* | 808 | 5.06656 | -74.84135 |
| Jabirú | *Eucometis penicillata* | 806 | 5.06656 | -74.84135 |
| Potosí | *Eucometis penicillata* | 788 | 3.40542 | -75.15874 |
| Jabirú | *Eucometis penicillata* | 773 | 5.06656 | -74.84135 |
| Boqueron | *Eucometis penicillata* | 769 | 4.27002 | -74.56168 |
| San Juan | *Eucometis penicillata* | 791 | 6.27975 | -74.10755 |
| San Juan | *Eucometis penicillata* | 1056 | 6.27975 | -74.10755 |
| Bateas | *Eucometis penicillata* | 1052 | 3.17081 | -75.25124 |
| San Juan | *Eucometis penicillata* | 1053 | 6.27975 | -74.10755 |
| San Juan | *Eucometis penicillata* | 1057 | 6.27975 | -74.10755 |
| Barbacoas | *Eucometis penicillata* | 803 | 6.71085 | -74.35172 |
| Potosí | *Eucometis penicillata* | 805 | 3.40542 | -75.15874 |
| San Juan | *Eucometis penicillata* | 767 | 6.27975 | -74.10755 |
| San Juan | *Eucometis penicillata* | 768 | 6.27975 | -74.10755 |
| Maceo | *Eucometis penicillata* | 785 | 6.55162 | -74.64276 |
| Río Manso | *Mionectes oleagineus* | NA | 5.66584 | -74.78178 |
| Maceo | *Leptopogon amaurocephalus* | NA | 6.55162 | -74.64276 |
| Maceo | *Leptopogon amaurocephalus* | NA | 6.55162 | -74.64276 |
| San Juan | *Eucometis penicillata* | NA | 6.27975 | -74.10755 |

**Appendix S2.** Statistical model to test for the effect of seasonality (dry vs. wet periods) on the probability of a bird to be infected. Seasonality categories were based on the sampling months for each individual.

Because our sampling was conducted across seasons (i.e., dry and wet periods), we evaluated whether the season in which individuals were caught affected infection probability. In the Magdalena River Valley, precipitation patterns are bimodal with dry seasons between December and February, and between June and August. Because we did not sample from October to December, the individuals caught in January, February, June – August were considered as dry season indviduals and those caught in March – May or September were considered as wet season individuals. We fitted a reduced parameter multinomial model in which the proportion of individuals caught in dry season *r* was considered independent from the proportion of indviduals infected *s* and compared the likelihood of the former model with a model in which the probabilities of capturing an individuals in the dry season and that the individual was infected were not independent from each other.

The probabilities for each of the four possible categories of the multinomial distribution were then defined as the proportion of individuals caught in the dry season and infected π_1=rs, caught in the wet season and infected π_2=(1-r)s, caught in the dry season and not infected π_3=r(1-s) and caught in the wet season and not infected π_4=(1-r)(1-s). We obtained the maximum-likelihood estimates of *r* and *s* by dividing the number of individuals caught in the dry season and the number of individuals infected by the total number of individuals captured respectively. We then compared the likelihood of the former model with a model in which the probabilities of capturing an individual in the dry season and that the individual was infected were not independent from each other. The maximum-likelihood estimates for the multinomial probabilities were given by the number of indviduals observed in each category divided by the total number of individuals caught. We then evaluated the goodness-of-fit of the model where infection probabilities are independent with a likelihood ratio test (similar to the one described for testing the influence of environmental variables on prevalence) using the equal probability infection model as null. We then estimated the confidence intervals of the MLEs using 1000 bootstrap replicates.

We found that the proportion of individuals caught in a season was independent from the proportion of individuals infected because the reduced-parameter multinomial fitted the data just as well as the fully parameterized model (*X*^2^ = 0.13, df=2, p=0.9). The proportion of individuals infected (s=0.15(0.1,0.18)) was lower than the proportion of individuals not infected (1-s = 0.85(0.82,0.9)) but the proportion of individuals was similar between seasons (r=0.53(0.5,0.6); π_1=0.077(0.05,0.1), π_2=0.068(0.05,0.07), π_3=0.45(0.41,0.47), π_4=0.4(0.33,0.48)).

**Appendix S3.** Pairwise correlations between variables for selection of independent variables to perform model evaluation. The comparisons in bold face indicate the variables that we considered to be highly collinear (i.e. R > 0.7). Precipitation: mean annual precipitation (2011-2014 dataset); Clouds: mean annual cloud frequency; Clouds.var: intra-annual cloud variability; PET: mean annual potential evapotranspiration; bio15: precipitation seasonality; bio17: precipitation seasonality (2011-2014 dataset).

|  | **Precipitation** | **Clouds** | **Clouds.var** | **PET** | **bio15** | **bio17** | **Spatial** | **Aridity** |
| --- | --- | --- | --- | --- | --- | --- | --- | --- |
| **Precipitation** | 1.00 |  |  |  |  |  |  |  |
| **Clouds** | 0.21 | 1.00 |  |  |  |  |  |  |
| **Clouds.var** | -0.32 | -0.58 | 1.00 |  |  |  |  |  |
| **PET** | **-0.84** | -0.31 | 0.48 | 1.00 |  |  |  |  |
| **bio15** | **-0.76** | 0.33 | -0.08 | 0.61 | 1.00 |  |  |  |
| **bio17** | **0.77** | -0.10 | -0.36 | **-0.82** | -0.62 | 1.00 |  |  |
| **Spatial** | **-0.90** | -0.02 | 0.35 | **0.90** | **0.73** | **-0.92** | 1.00 |  |
| **Aridity** | **0.99** | 0.22 | -0.30 | **-0.85** | **-0.71** | **0.79** | **-0.91** | 1.00 |

**Appendix S4.** Loadings and cumulative proportion of variance explained resulting from the principal component constructed to reduce the independent variables used to explain the variability on haemosporidian parasites along the rainfall gradient of the Magdalena Valley. Precipitation: mean annual precipitation (2011-2014 dataset); Clouds: mean annual cloud frequency; Clouds.var: intra-annual cloud variability; PET: mean annual potential evapotranspiration; bio15: precipitation seasonality; bio17: precipitation seasonality (2011-2014 dataset).

| **Variable** | **PC1** | **PC2** | **PC3** |
| --- | --- | --- | --- |
| *Precipitation* | 0.46 | 0.04 | -0.2 |
| *Clouds* | 0.09 | -0.71 | -0.53 |
| *Clouds.var* | -0.25 | 0.51 | -0.66 |
| *PET* | -0.45 | 0.07 | 0.04 |
| *bio15* | -0.35 | -0.46 | 0.14 |
| *bio17* | 0.42 | 0.1 | 0.39 |
| *Aridity* | 0.46 | 0.01 | -0.26 |
| *Cumulative Proportion* | 0.63 | 0.85 | 0.93 |

**Appendix S5.** Results from models explaining the variability on haemosporidian parasites along the rainfall gradient of the Magdalena valley obtained using the three principal components and species identity as independent variables.

| **Model** | **BIC** | **ΔBIC** |
| --- | --- | --- |
| Species | 89.81 | 0.00 |
| Species + PC1 | 91.94 | 2.14 |
| Species + PC3 | 92.03 | 2.23 |
| Species + PC2 | 92.89 | 3.08 |
| Species + PC1 + PC3 | 93.77 | 3.96 |
| Species + PC1 + PC2 | 95.32 | 5.51 |
| Species + PC2 + PC3 | 95.52 | 5.71 |
| Species + PC1 + PC2 + PC3 | 97.53 | 7.72 |
| Intercept | 132.89 | 43.08 |
| PC2 | 135.09 | 45.28 |
| PC1 | 136.15 | 46.35 |
| PC3 | 136.78 | 46.97 |
| PC1 + PC2 | 138.71 | 48.91 |
| PC2 + PC3 | 139.01 | 49.20 |
| PC1 + PC3 | 140.01 | 50.20 |
| PC1 + PC2 + PC3 | 142.65 | 52.84 |

Appendix S6. Coefficient of variation (CV) of the variables considered for the analyses and Temperature. PET= Potential Evapotranspiration, Clouds = Mean annual cloud cover, Clouds Var = Intraannual variability in cloud cover, Bio15 = Precipitation seasonality, Bio17 = Precipitation of the driest quarter.


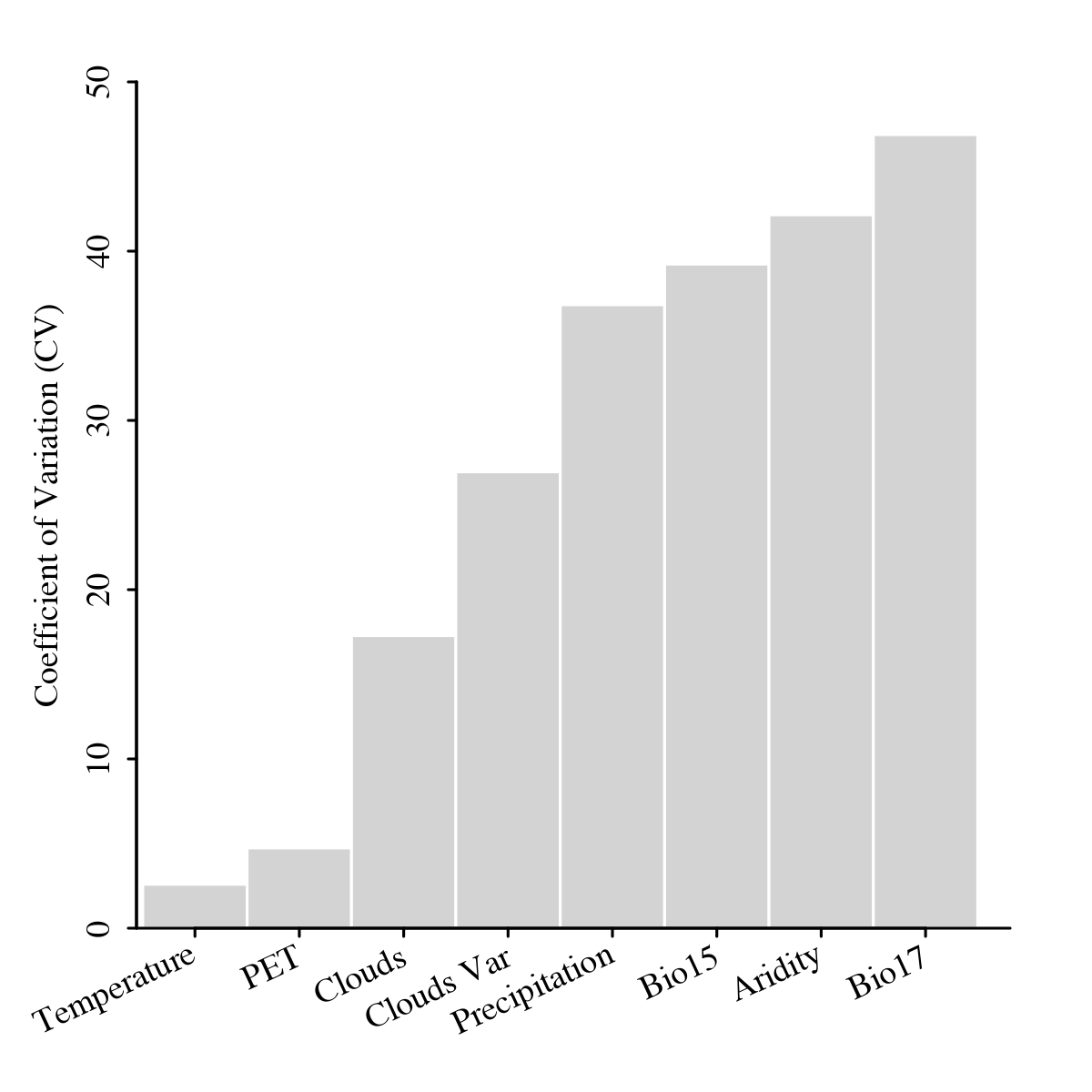

Supplement: Supplementary file 1 [file ECE3-8-3800-s001.docx]
